# Hypertension From the Perspective of Iranian Traditional Medicine

**DOI:** 10.5812/ircmj.16449

**Published:** 2014-03-05

**Authors:** Roshanak Ghods, Manouchehr Gharooni, Gholamreza Amin, Esmaeil Nazem, Alireza Nikbakht Nasrabadi

**Affiliations:** 1 College of Traditional Medicine, Tehran University of Medical Sciences, Tehran, IR Iran; 2 College of Traditional Medicine, Iran University of Medical Sciences, Tehran, IR Iran; 3 School of Medicine, Tehran University of Medical Sciences, Tehran, IR Iran; 4 Department of Pharmacognosy, Faculty of Pharmacy, Tehran University of Medical Sciences, Tehran, IR Iran; 5 School of Nursing and Midwifery, Tehran University of Medical Sciences, Tehran, IR Iran

**Keywords:** Hypertension, Medicine, Traditional, Iran

## Abstract

**Background:**

Nowadays, hypertension is considered as a global public health issue and in recent decades, it has shown a growing trend due to changes in lifestyle.

**Objectives:**

The purpose of this investigation was to compare symptoms of hypertension with known diseases in ancient medical texts and to find a disease that had the maximum overlap of symptoms with hypertension.

**Materials and Methods:**

In this qualitative study, reliable sources of traditional medicine such as The Canon of Medicine by Avicenna, The Complete Art of Medicine (Kitab Kamil as-Sina’aat-Tibbiyya) by Haly Abbas, Facilitating Treatment and a letter for Health preservation (Tahsil Al-Elaj and Resale Hafez Al-Sehha) by Mohammad Taghi Shirazi, and some reliable resources of conventional medicine such as Harrison’s principles of internal medicine and databases such as Pubmed, Scopus, SID, and Magiran were probed base on keywords to find a disease that had the most overlapping symptoms with hypertension. By taking notes from the relevant materials, the extracted texts were compared and analyzed.

**Results:**

Findings showed that hypertension has the most overlap with Imila (accumulation of normal or abnormal fluid in the body) symptoms in Iranian traditional medicine. Although this is not a quietly perfect overlap and there are other causes and reasons including dry dystemperament of vessel wall (atherosclerosis), hot dystemperament of heart or damages to other organs like liver, kidney and nervous system that could also lead to hypertension according to Iranian traditional medicine.

**Conclusions:**

Finding the equivalent disease to HTN based on Iranian traditional medicine, could suggest a better strategy for preventing, treating and reducing debilitating its complications in the future. In conclusion, we can approach to hypertension with recommendations for reducing Imtila when we are dealing with a kind of hypertension that corresponds to Imtila. Therefore, if patient is suffering from another type of hypertension like dry dystemperament of vessel wall, it surely requires another treatment approach for reducing vessel wall dryness.

## 1. Background

Hypertension (HTN) is one of the established and modifiable risk factors associated with cardiovascular diseases (CVD) (1). The prevalence of HTN increases with age to the extent that one out of every two individuals older than 60-year-old is suffering from high blood pressure (2). According to health statistics issued by the World Health Organization (WHO) in 2012, the global prevalence rates of HTN (systolic blood pressure (SBP) ≥ 140, diastolic blood pressure (DBP) ≥ 90) in males and females aged ≥ 25 years were 29.2% and 24.8%, respectively (3). Almost 30% of American adults are affected by HTN (4). There has been a rapid increase in the prevalence rate of HTN in India, China, Philippines, Thailand, Sri Lanka, Pakistan, Nepal and Iran. HTN prevalence (BP > 140/90 mmHg) varies between 15% to 35% in urban adult populations of Asia. In rural populations, the prevalence rate is two to three times lower than in urban areas (5). According to a survey conducted by Esteghamati et al. the prevalence of HTN in Iranian population aged between 15 and 64 years was 26.6 % (6).

In addition to the high as well as growing prevalence of the disease, increased blood pressure doubles the risk of CVD including coronary artery disease (CAD), congestive heart failure (CHF), ischemic or hemorrhagic stroke, kidney failure, and peripheral vascular disease (7). Since most cases of HTN cannot be conclusively cured (8), the control of blood pressure requires lifelong treatment with drugs that are costly and might cause side effects. Sometimes, these side-effects might be more problematic than HTN itself (9). Given the importance of this disease, WHO has announced the slogan for The World Health Day of 2013 as “healthy heart beat, healthy blood pressure”. Therefore, addressing this disease from the viewpoint of other medical schools like Iranian traditional medicine, which is one of the richest ancient medical schools, might provide a better understanding of this “silent killer”.

## 2. Objectives

Since HTN was not mentioned with the same phrase or its equivalents in ancient medical texts, the purpose of this study was to compare the symptoms of HTN with known diseases in ancient medical books and to find a disease that has the maximum overlap of symptoms with HTN. Finding the equivalent disease might suggest a better strategy for preventing, treating, and reducing the debilitating complications of HTN in the future.

## 3. Materials and Methods

In this qualitative study, traditional resources of different ages, such as The Canon of Medicine by Avicenna (980–1037 AD), The Complete Art of Medicine (KitabKamil as-Sina’aat-Tibbiyya) by Haly Abbas (949–982 AD), and Facilitating Treatment and a Letter for Health Preservation (Tahsil Al-Elaj and Resale Hafez Al-Sehha) by Mohammad Taghi Shirazi (1814-1896) were firstly selected according to expert panel of judge. Likewise, Harrison’s Principles of Internal Medicine (the main textbook of internal medicine) and databases like Pubmed, Scopus, and some Iranian databases like SID and Magiran were searched based on keywords in order to extract evidences and to find probable HTN symptoms. Then, related materials were compared and summarized to find diseases that might have the greatest overlap of symptoms with clinical manifestations of HTN.

All data were discussed in lots of research sessions including reading and reviewing the original data, clarifying the issues that were not clear in the translated quotations, and further interpretations of additional parts when needed. The condensed meaning units, which were extracted by the first author, were shared with the research group that considered all the material along with questioning and discussing the whole content. All authors were involved in the process of analysis. The trustworthiness criteria of qualitative research were carefully followed to ensure the study’s rigor. The interpretation was further discussed in regular meetings with the research team. All analyses of the texts were compared and contrasted. Furthermore, the research team continuously consulted with each other to deal with any ambiguities in the analysis process and to select clear and precise words and phrases for labeling themes.

## 4. Results

### 4.1. Definition and Symptoms of Hypertension in Terms of Conventional Medicine

Blood pressure is the force of blood against the walls of arteries. According to the internal medicine resources, HTN has no clinical manifestations in the majority of cases to the extent that has been named as the “Silent Killer” (10). If clinical symptoms led the patient to consult with a doctor, these symptoms generally fall into three categories:

1. Symptoms of rising of blood pressure (occipital headache, dizziness, palpitation, etc.)
2. Symptoms due to vascular diseases (hematuria, vision problem, dyspnea, etc.)
3. Symptoms of the underlying diseases causing secondary HTN (9).

### 4.2. Comparison of Hypertension Manifestations With the Diseases in Ancient Medical Texts

#### 4.2.1. Production of Proper and Improper Humors in the Body

Based on the principles of traditional medicine, the eaten food passes through various stages of digestion before reaching to the organs. Avicenna and most scholars are of the opinion that digestion is a continuous process that occurs from mouth to organs, therefore, it can be divided into four successive stages of gastric, hepatic, vascular, and organic digestions. At each stage of digestion, the eaten food changes to become suitable to use by the body. In each digestion process, the following actions occur:

In gastric digestion, some aspects and features of foodstuffs are altered and suitable absorbent materials called “Chilos” are absorbed into the liver via the mesenteric artery for further digestion. Feces are considered as the waste material of this digestion. In hepatic digestion, “Chilos” changed into “Chymus” which composed of four humors of (blood, phlegm, yellow bile, and black bile) and flows into the vessels. Here, the waste matter is urine. In vascular digestion, the food situation gets closer to the organ form and in organic digestion, the food is completely similar to the target organ tissue. The waste material is sweat (11). Therefore, humors are the final product of liver digestion and in order to have good quality, two things must occur:

- Normal function of the stomach causing production of the appropriate gastric emulsion in order to form high quality humors in the liver.
- Normal function of the liver for proper digestion (11, 12).

#### 4.2.2. Consequence of Improper Humors Production

If rotten foods enter into the stomach, and if the digestive power of gastrointestinal tract act properly, this food would hurt neither the stomach nor its adjacent organs. In this case, the stomach digests the rotten foods and produce a spoiled sap (gastric digestion) that would enter into second step of digestion (hepatic digestion); after entering the liver, it produces low quality blood and improper humors that would penetrate into all vessels and organs of body. Following penetration into the vessels and body organs, two states occur:

- If the body nature and expulsive force of organs were strong, waste materials and unrighteous humors would be disposed and sent beneath the skin that might lead to the various skin problems such as boils, pimples, and skin eruptions.
- If the expulsive force of an organ was weak and unable to repel the bad humors, these humors would cause disease in the respective organ. For example, existence of unrighteous humors, blood, and accumulation of waste products in the head and their increase in the arteries, might cause sanguine stroke and nasal bleeding (13).

From the viewpoint of traditional medicine, in the case of overindulgence in eating and gastric Imtila, stomach cannot properly digest the food. The improper digested food enters into the arteries and remains undigested and raw in vessels. Considering that there is still digestion in the vessels, although much weaker in comparison to the gastric digestion, undigested food could not be fully digested in vessels, as well. Penetration of undigested and raw materials into vessels causes symptoms such as feeling of heaviness, lethargy, body stretching, and yawning in patient. In severe cases, excessive fullness of the blood vessels with raw materials can cause fatal side effects such as arterial wall stretching or tearing (13). With reference to the above description, it seems that HTN originates from Imtila, especially gastric Imtila, which is the source of almost all diseases (14-16). Therefore, we compared the various signs and symptoms of Imtila with HTN (Box 1).

**Box 1.** Comparison of Hypertension and Imtila Clinical Features

Hypertension (9)	Imtila (12, 17)
**Early morning occipital headaches**	Heaviness in the head and headache (18)
**Easy fatigability, muscle weakness**	Fatigue, lethargy and feeling of heaviness (18)
**-**	Exhaustion, flaccidity of the body organs and loose motions (18)
**Blurred vision due to retinal lesions (Vision problems)**	Blurred vision
**Hematuria**	Turbid urine (18)
**Aortic dissection or aneurysm leak**	Prominent, distended and tense vessels which increased risk of rupture (18)
**Transient ischemic attack (Ischemic and hemorrhagic strokes are common complications of hypertension)**	Sanguinary stroke (18)
**Flushing is one of the neurogenic hypertension or pheochromocytoma symptoms (19, 20)**	General redness
**Epistaxis (21, 22)**	Nasal bleeding
**Change in skin elastic fibers and vascular structure (Rarefaction phenomenon) (23-26)**	Skin tension
**Erratic sleep has been cited in evidence of secondary hypertension (9)**	Dreams with the subject of heaviness in such a way that person dreams of inability to move, stand up, speak, or bearing a heavy burden.
**Anorexia as the symptoms of heart failure that is considered as a complication of hypertension (9)**	Loss of appetite
**-**	Hypervolemia pulse/ Fullness of Pulse (Nabz mumtali)
**Palpitation**	-
**Dyspnea**	-
**Impotence**	-

Lack of complete overlap of known symptoms of HTN with Imtila confirms that increased blood pressure can occur due to other causes and reasons as well; therefore, these symptoms will not completely match. Hence, it does not make sense to consider HTN and Imtila as exact synonymous as it might lead to ignore other causes of HTN including vessel wall dry dystemperament (Sui’ a Mizaj) caused by descending of abnormal black bile (abnormal Sauda) to vessel wall, which seems to be equivalent to atherosclerosis (27, 28), hot dystemperament of the heart, or illnesses associated with organs such as nervous system, liver, and kidneys.

## 5. Discussion

Findings revealed that HTN would roughly be considered as an equivalent to Imtila although they are not exactly the same. This would be more understandable by defining the exact concept of Imtila and its different types.

### 5.1. Definition of "Imtila"

Literally, ‘Imtila’ means fullness of the body with fluids. Technically, Imtila means accumulation of normal or abnormal fluids in the body (29). In the past, physicians used the term of Imtila or congestion to describe a condition in which fluids accumulated in the body producing certain symptoms (18). These materials could fill free spaces inside tissues and ducts; For example, they might cause obstruction in vessels and result in infarction. Obstruction of vessels especially arteries and in particular arteries of organs such as heart, brain, and liver is extremely dangerous (30). Rhazes, Avicenna, and Haly Abbas described the Imtila in this way that excess of food, alcohol, and rest in addition to lack of exercise would result in accumulation of waste products in the body, whether beneficial (Mahmooda) or Non-beneficial (Ghair-mahmooda), both would be toxic for the body. The accumulation of these waste products might lead to increase in blood volume, vessels wall tension, and vascular pressure (29).

### 5.2. Types of Imtila

Scholars had mentioned the types of Imtila as: “Imtila bi hasbil auiya” (fullness of body channels) and “Imtila b’hasb-ul-quva” (inadequacy of natural body force to digest) (29). The “Imtila bi hasbil auiya” develops when there is excess accumulation of matter (madda), blood (dam), and essence (rooh) in the vessels leading to their distension (tamaddud) and tension (18). According to some ancient texts, “Imtila bi hasbil auiya” is somewhat synonymous with Sanguine Imtila (12, 17). In this situation, exuberance of Chymus inside veins and arteries leads to distension and tension of vessels. This kind of Imtila can be considered as change in quantity of blood or increased intravascular volume.

“Imtila b’hasb-ul-quva” develops when quality of matter changes besides retention of non-beneficial humors due to weak body power (18). In this situation, the body power weakens, loses its ability to digest and mature materials, and cannot transport wastes and improper humors. This kind of Imtila can be considered as change in quality of blood. Increased blood viscosity is an example of blood quality change due to its mixture with improper humors. Based on above definitions, it can be inferred that HTN can be synonymous to Imtila only in the case of changes in blood quantity or quality. In fact, HTN in terms of change in blood quantity is roughly corresponding to "Imtila bi hasbil auiya" and from the perspective of qualitative change, is approximately corresponding to "Imtila b’hasb-ul-quva" in Iranian traditional medicine.

Farooq Ahmad Iqbal in his treatise considers the clinical features of Imtila corresponding to the clinical features of HTN; and therefore, Imtila and HTN can be safely referred to the same condition. Furthermore, he claims that the term “Zaghtuddam Qavi” was adopted as translation of the term “HTN in Unani System of Medicine (18). Likewise, Shamshad Ahmad considers that the term “Zaghtuddam Qavi” did not exist with such a definition in the classical Unani literature and most of HTN symptoms were mentioned under the heading “Imtila bi hasbil auiya”. He also believes that symptoms mentioned by Rhazes in description of this type of Imtila are similar to clinical features of HTN (29). From the viewpoint that considers HTN and Imtila as corresponding entities, these studies endorse the findings of current study. However, based on principles of Iranian traditional medicine, increase in blood pressure is not exactly Imtila and might also occur due to other etiologies such as vessel wall dry dystemperament, impaired cardiac pump due to hot dystemperament of heart, or even involvement of other organs outside the cardiovascular system including kidneys, liver, and nervous system while no change has been occurred in quality or quantity of circulating blood. For example, descending of black bile in vessel wall or so-called atherosclerosis along with reducing elasticity of arteries would lead to HTN in spite of normal blood volume or suitability of blood quality (27, 28). Meanwhile, quoting Rhazes, Faruq Ahmad Iqbal considers development of "Imtila bi hasbil auiya" as a result of eating inappropriate foodstuffs that leads to production of improper humors in the body (18). This is consistent with findings of our study that considered gastric Imtila and fullness of body ducts and vessels as the origin of almost all kind of Imtila diseases. This study was performed on a limited number of available resources of Iranian traditional medicines. There was remarkable limitations regarding availability of related and appropriate information about Iranian traditional medicine thorough internet searches in scientific data banks; therefore, most searches had to be done manually thorough available old books. In addition, the nature of study limited the ability to generalize the results. However, as with all qualitative studies, results were not intended to be generalized. Since HTN was considered synonymous to Imtila according to quantitative and qualitative changes of blood in ancient medical text, we must first check for symptoms and manifestations of Imtila in dealing with each patient. Thereafter, if the patient has majority of aforementioned diagnostic criteria, he/she would be treated based on nutritional or medical guidelines and instructions listed under the category of Imtila in traditional medicine textbooks. It is obvious that if patient has few of the mentioned symptoms and he/she has been diagnosed to be suffering from other causes like dry dystemperament of vessel, treatment approach would definitely be different and instead of concentration on Imtila, appropriate treatment should be chosen for curing vessel dryness until the most therapeutic response is achieved. This is also true about other possible causes of HTN such as hot dystemperament of heart and involvement of other organs associated with the cardiovascular system including liver, kidneys, and nervous system. Each of these requires its own treatment that can be different from treatment of HTN caused by types of Imtila.
